# Effect of Phosphate Addition to Electrolytes on Corrosion Behavior of Stainless Steels in Seawater Electrolysis

**DOI:** 10.1021/acsomega.5c11989

**Published:** 2026-03-29

**Authors:** Tomoya Hashimoto, Mariko Kadowaki, Yoshiharu Murase, Masaya Shimabukuro, Kazuhiro Takanabe, Masakazu Kawashita, Hideki Katayama, Yusuke Tsutsumi

**Affiliations:** † 52747 National Institute for Materials Science, 1-2-1 Sengen, Tsukuba, Ibaraki 305-0047, Japan; ‡ Laboratory for Biomaterials and Bioengineering, Institute of Integrated Research, 538642 Institute of Science Tokyo, 2-3-10 Kanda-Surugadai, Chiyoda-ku, Tokyo 101-0062, Japan; § Department of Chemical System Engineering, School of Engineering, 13143 The University of Tokyo, 7-3-1 Hongo, Bunkyo-ku, Tokyo 113-8656, Japan

## Abstract

Water electrolysis is an effective method for producing hydrogen, which is a valuable alternative to fossil fuels. However, the presence of chloride ions can cause significant structural degradation, and electrolysis systems compatible with seawater must be developed. In this study, phosphate was added to an electrolyte simulating seawater electrolysis, and the effects on the corrosion resistance of ferritic (Type 430) and austenitic (Type 304 and 316) stainless steels were investigated using electrochemical techniques. The phosphate-induced changes in the passive films on the steels were examined using X-ray photoelectron spectroscopy (XPS). The phosphate in the electrolyte enhanced the pitting corrosion resistance of all the stainless steels. However, excessive phosphate concentrations promoted the partial dissolution of the passive film, particularly for the 430 steel. XPS analysis showed that phosphorus was incorporated into the passive film as phosphate for every type of steel in this study, which likely enhanced the pitting corrosion resistance. Cyclic polarization measurements of the 430 steel indicated that the pH-buffering action of the phosphate in the electrolyte suppressed pitting propagation. These findings provide fundamental insights into the role of phosphate additives in stabilizing stainless steels, which may contribute to the improved safety of seawater electrolysis systems.

## Graphical abstract

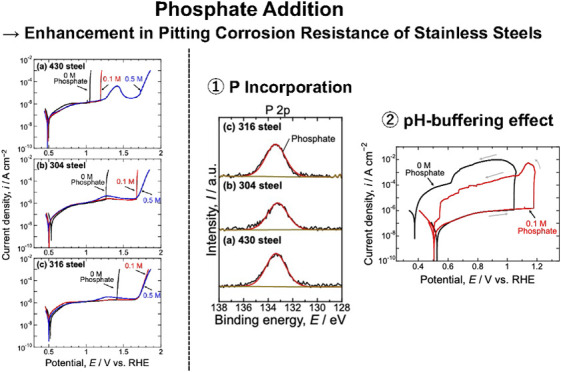

## Introduction

Hydrogen is a valuable alternative to fossil fuels that is crucial for reducing carbon dioxide emissions. Water electrolysis is an effective method for producing hydrogen by electrically splitting water into hydrogen and oxygen.^1–4^ Various electrolysis systems have been developed, including alkaline water, proton exchange membrane, and anion exchange membrane electrolysis systems, some of which are already in practical use.^3,4^ However, current systems require high-purity water free from chloride ions (Cl^–^) to prevent degradation caused by corrosion. However, the majority of the water resources on Earth are seawater; therefore, electrolysis systems that utilize seawater or quasi-native seawater must be developed for low-cost and efficient hydrogen production.^5^ Seawater electrolysis can also utilize abundant renewable energy sources such as wind and solar energy. Consequently, seawater electrolysis has attracted considerable attention and has been the subject of numerous studies.^6^

The development of low-cost water electrolysis systems requires materials that are cost-effective with satisfactory mechanical properties, particularly for electrochemical cells, which constitute a large part of the system. Ferrous alloys, including stainless steels, are desirable because they are inexpensive and have excellent mechanical properties, and they are expected to be applied in cell frames and electrodes.^7–10^ However, seawater contains a high concentration of Cl^–^, which promote corrosion. Therefore, corrosion degradation is a major challenge in seawater electrolysis. Moreover, hypochlorite (ClO^–^) or chlorine gas (Cl_2_) is generated at the anode through the chlorine oxidation reaction, depending on the pH (Cl^–^ + H_2_O ⇌ ClO^–^ + 2H^+^ + 2e^–^, or 2Cl^–^ ⇌ Cl_2_ + 2e^–^, respectively).^11^ This leads to the formation of hypochlorous acid, which is a strong oxidizing agent that further accelerates corrosion. Therefore, in harsh environments that contain large amounts of Cl^–^, stainless steels are susceptible to pitting corrosion.^12,13^

Adding phosphate not only improves the hydrogen evolution reaction (HER) performance^14^ but also effectively suppresses corrosion of the metallic materials.^15–17^ Komiya et al. conducted electrochemical measurements using NiFeO_*x*_/Ni electrodes in electrolytes containing both phosphate and chloride, and they found that phosphate suppressed Ni dissolution and subsequent corrosion deterioration.^18^ Kadowaki et al. analyzed the mechanism of this corrosion suppression and reported that, in an electrolyte containing phosphate, phosphorus was incorporated into the Ni passive film, which changed the passive film structure and improved corrosion resistance.^19^ In addition to Ni-based materials, studies on phosphate manufacturing plants and fuel cells have suggested that adding phosphate to an electrolyte may enhance the corrosion resistance of stainless steels.^20–22^ For example, Salah et al. investigated the corrosion behavior of Sanicro28 austenitic stainless steel in a 50 wt % H_3_PO_4_ solution, and they revealed that the corrosion resistance improved as the immersion time in a phosphate-containing solution increased.^21^ Wang et al. also reported that austenitic 316L, 317L, and 904L stainless steels exhibited effective passivation in a 98% H_3_PO_4_ solution at 170 °C.^22^ Moreover, Munis et al. investigated the effect of adding phosphate on corrosion resistance of type 316L austenitic stainless steel in chloride contaminated simulated coal gasification wastewater environment.^23^ They found that the phosphate plays a role in improving the corrosion resistance.

These insights could apply to realize for seawater electrolysis systems. However, previous studies have focused on the corrosion behavior of stainless steels in acidic solutions with very low Cl^–^ concentrations, where they suffer uniform corrosion. By contrast, very few studies have analyzed the effects of phosphate in near-neutral-pH environments containing high concentration of Cl^–^ (i.e., seawater electrolysis environments). In these environments, corrosion mainly occurs as pitting. However, the effects of phosphate on pitting corrosion resistance remain unclear. Moreover, most previous studies on phosphate addition have focused on austenitic stainless steels. Ferritic stainless steels, which contain no Ni, are attractive alternatives owing to their low production cost. However, no previous studies have investigated the effects of adding phosphate to the electrolyte on the corrosion resistance in the absence of Ni-based reinforcement mechanisms.

This study aim of investigating of the effect of phosphate on changes in corrosion resistance of stainless steels in seawater electrolysis environments for hydrogen production, characterized by the conditions (high Cl^–^, phosphate concentrations and pH 9.2). The effect of phosphate on austenitic (type 304, 316) and ferritic (type 430) stainless steels was systematically evaluated. The stainless steels are general grades, and desirable to utilize for the components of seawater electrolysis systems, such as electrochemical cells and electrodes. Polarization measurements of the stainless steels were conducted in the electrolytes various concentrations of phosphate to assess the effects of phosphate on the corrosion resistance. Furthermore, the mechanism by which phosphate improved the corrosion resistance was discussed in terms of the change in the passive film properties and the pH-buffering effect of phosphate. The changes in the passive film were analyzed using X-ray photoelectron spectroscopy (XPS), and the pH-buffering effect was examined using cyclic polarization measurements.

## Experimental Section

### Materials and Solutions

The 430, 304, and 316 steel plate samples were purchased from Nilaco Corporation. The chemical compositions provided by the manufacturer are presented in Table 1. Each sample was cut into approximately 2 cm square specimens, mechanically ground using a series of SiC papers, and then polished sequentially with 6 and 1 μm diamond pastes. The specimens were then cleaned with ethanol.

**Table 1:** Chemical Compositions (Mass%) of the 430, 304, and 316 Steel Samples

steel type	C	Si	Mn	P	S	Ni	Cr	Mo	Co	Fe
430	0.02	0.15	0.75	0.03	0.004	0.15	16.25	-	-	Bal.
304	0.07	0.53	1.21	0.033	0.004	8.16	18.28	-	0.3	Bal.
316	0.04	0.63	0.91	0.038	0.004	10.21	16.81	2.07	0.22	Bal.

For the electrochemical measurements, the composition and pH of the electrolyte were determined based on previous studies.^18,19^ The electrolytes consisted of 0.5 M K-borate; 0.5 M KCl; and 0, 0.1, or 0.5 M phosphate (pH 9.2). H_3_BO_3_ (>99.5%, Kanto Chemical, Japan), KCl (>99.5%, Kanto Chemical, Japan), and H_3_PO_4_ (≥85%, FUJIFILM Wako Pure Chemical, Japan) were dissolved in deionized water to obtain the desired concentrations. Then, the pH was adjusted to 9.2 using a small amount of 15 M KOH (≥85%, FUJIFILM Wako Pure Chemical, Japan).

### Electrochemical Measurements

Potentiodynamic polarization of specimens in 0.5 M H_3_BO_3_–0.5 M KCl–*x* M H_3_PO_4_ (*x* = 0, 0.1, or 0.5) was conducted using electrochemical measurement systems (Hz-7000 and Hz-Pro S12, Meiden Hokuto Corp., Japan) at room temperature without stirring under naturally aerated conditions. A conventional three-electrode system was used in this study (Figure 1), with the specimen, a Pt wire, and a commercial Ag/AgCl electrode (RE-T7A, EC FRONTIER CO., Ltd., Japan) in saturated KCl as the working, counter, and reference electrodes, respectively. Hereinafter, all the potentials are described with respect to a reversible hydrogen electrode (RHE). For each text, the exposed surface area of the specimen was restricted to a 1 cm^2^ circle at the bottom of the cell. The specimen was held at the open circuit potential (OCP) for 30 min. After confirming that the OCP value had stabilized, the potential was scanned anodically at a rate of 20 mV min^–1^, starting at 50 mV below the final OCP, until the current density reached 1 mA cm^–2^. The pitting potential was defined as the potential at which the current density reached 100 μA cm^–2^. Cyclic polarization measurements of the 430 steels in the electrolyte were used to assess the pH-buffering effect of the phosphate. The potential was first scanned anodically, as described above. When the current density reached 1 mA cm^–2^, the potential sweep direction was reversed at the same scanning rate until the current density reached −1 μA cm^–2^.

**Figure 1:**
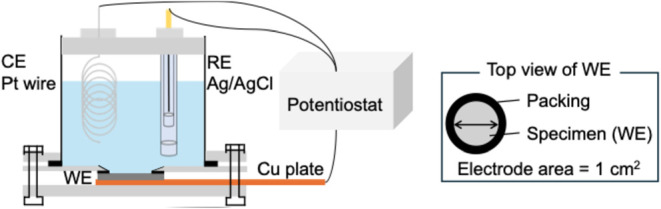
Schematic illustration of the electrochemical cell.

### Surface Analysis

The surface morphologies of the specimens were observed using an optical microscope (VHX-5000, Keyence, Japan). The passive films on the specimens were analyzed using XPS (JPS-9010MC, JEOL, Japan). All the binding energies reported in this paper are relative to the Fermi level, and all the spectra were obtained using the Al Kα line (*E* = 1486.6 eV). The measurements were performed in the narrow scan mode (pass energy of 20 eV). The spectrometer was calibrated using Au, Ag, and Cu. The background was subtracted from the measured spectrum using Shirley’s method.^24^ The Lorentzian–Gaussian model was used for peak fitting, and all the peaks were corrected to the C 1s binding energy (285.0 eV). The composition and thickness of the surface oxide film were calculated simultaneously using methods described in previous studies.^25,26^ Empirical data^27,28^ and theoretically calculated data^29^ for the relative photoionization cross sections were used for quantification.

## Results and Discussion

### Effects of Phosphate on the Pitting Corrosion Resistance of Stainless Steels

The effects of phosphate on the corrosion resistance of stainless steels were investigated by conducting potentiodynamic anodic polarization measurements in 0.5 M K-borate–0.5 M KCl electrolytes containing 0, 0.1, or 0.5 M phosphate (pH 9.2). Figure 2 shows the polarization curves for the 430, 304, and 316 steel specimens. Considering the 430 steel (Figure 2a), below 1.0 V_RHE_, the anodic current did not increase rapidly in any of the electrolytes, and the current density remained below 10^–6^ A cm^–2^, indicating that the specimen surfaces were well-passivated. In the electrolyte without phosphate, a rapid logarithmic increase in the anodic current, indicative of typical pitting corrosion behavior, was observed at approximately 1.1 V_RHE_. Optical microscopy of the sample after polarization confirmed the occurrence of pitting corrosion, as shown in Figure 3a-1. In the electrolyte containing 0.1 M phosphate, a similar logarithmic increase in anodic current was observed at 1.2 V_RHE_, and pitting corrosion was also evident after polarization (Figure 3a-2). This indicates that the addition of 0.1 M phosphate slightly increased the pitting potential. When the phosphate concentration was increased to 0.5 M, a slight increase in the anodic current was observed at approximately 1.4 V_RHE_, and the logarithmic increase observed in the electrolytes without phosphate and with 0.1 M phosphate was absent. The anodic current increased significantly when the potential exceeded 1.7 V_RHE_, which was attributed to the OER and/or transpassive dissolution, rather than pitting. In this case, only small pits were observed in the optical microscopy image (Figure 3a-3). Therefore, the predominant corrosion morphology of the 430 steel was pitting, and the addition of phosphate to the electrolyte enhanced the pitting corrosion resistance. Under the conditions of this study, only pitting corrosion was observed; however, it is note that crevice corrosion may also occur in practical electrochemical cells.

**Figure 2:**
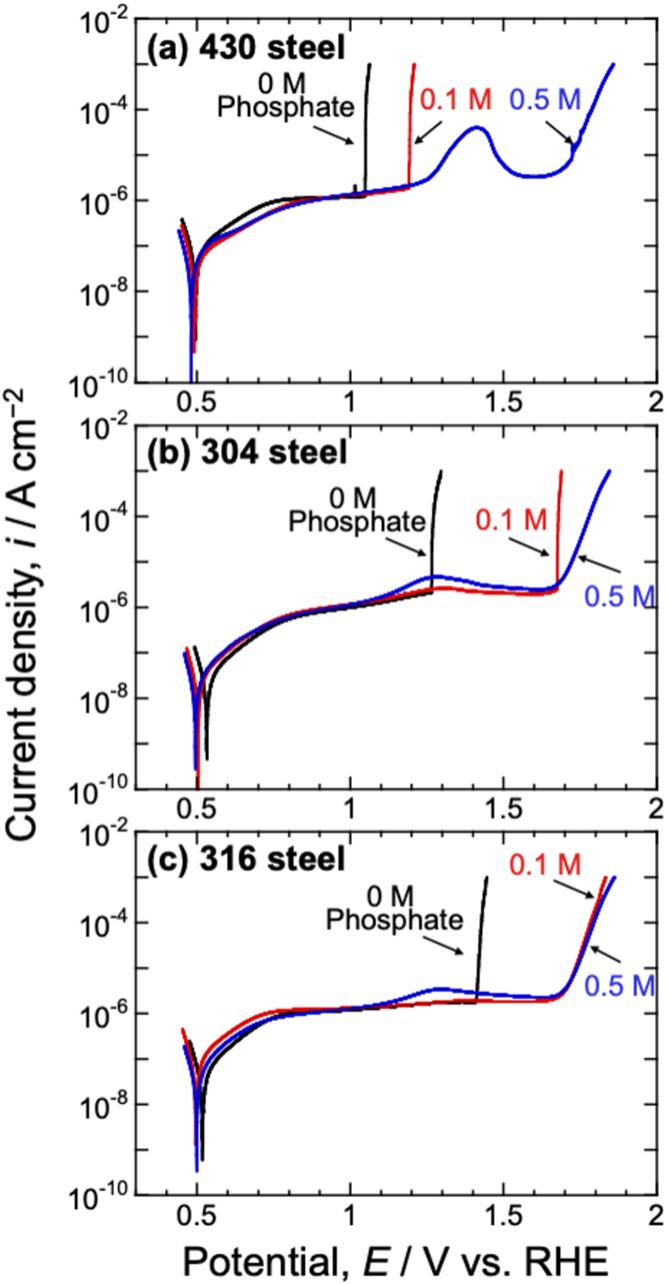
Potentiodynamic polarization curves for (a) 430, (b) 304, and (c) 316 steels in electrolytes consisting of 0.5 M K-borate, 0.5 M KCl, and 0, 0.1, or 0.5 M phosphate at pH 9.2. The measurements were performed at room temperature.

**Figure 3:**
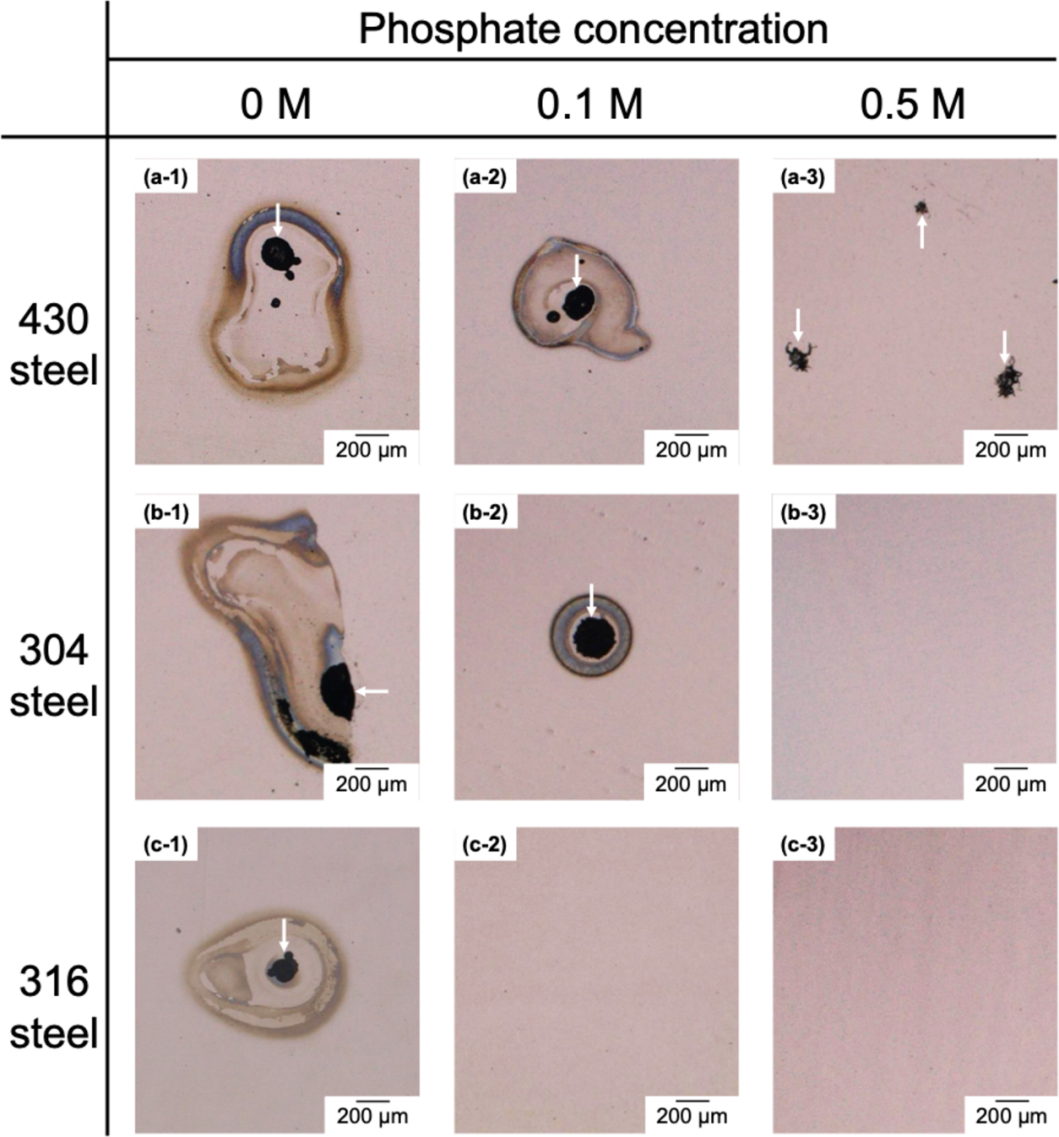
Optical microscopy images of (a) 430, (b) 304, and (c) 316 steels after anodic polarization in the electrolytes consisting of 0.5 M K-borate, 0.5 M KCl, and 0, 0.1, or 0.5 M phosphate at pH 9.2. The arrows indicate pits present on the surface.

Similarly, the addition of phosphate enhanced the pitting corrosion resistance of both 304 and 316 steels. In the electrolyte without phosphate, the 304 and 316 steels both showed rapid logarithmic increases in the anodic current (Figure 2b,c, black lines) and pitting corrosion (Figure 3b-1 and c-1). In the electrolyte containing 0.1 M phosphate, the pitting potential of the 304 steel shifted in the positive direction, although pits were still observed. By contrast, the 316 steel did not show a rapid logarithmic increase in the anodic current, which increased gradually above approximately 1.7 V_RHE_. Moreover, no pitting corrosion was observed on the 316 steel (Figure 3b-2). In the electrolyte containing 0.5 M phosphate, the 304 and 316 steels did not show logarithmic increases in the anodic current or pitting corrosion (Figure 3b-3 and c-3).

Figure 4 shows the pitting potentials of the 430, 304, and 316 steel specimens as a function of the phosphate concentration. The pitting potential was defined as the potential at which the anodic current reached 100 μA cm^–2^, and the given values represent the average of three measurements. The error bars for each condition indicate the standard deviation of the three measurements. For simplicity, even in cases where no pitting corrosion was observed in Figure 2 (i.e., 430 and 304 steels with 0.5 M phosphate, and 316 steel with 0.1 and 0.5 M phosphate), the data are indicated with arrows. In the absence of phosphate, the 430 steel exhibited the lowest pitting potential, followed by the 304 and 316 steels. With the addition of phosphate, a positive shift in the pitting potential was observed for all the steel types. The phosphate addition to the electrolyte was found to be more effective at enhancing corrosion resistance in austenitic stainless steels than in ferritic steel. In our previous study, we focused on pure Ni and demonstrated that phosphate in the electrolyte engages in electrochemical interactions with Ni, improving its passivity.^19^ Thus, this is considered to be due to interaction between phosphate and Ni, which is present only in austenitic steels, leading to enhanced corrosion resistance.

**Figure 4:**
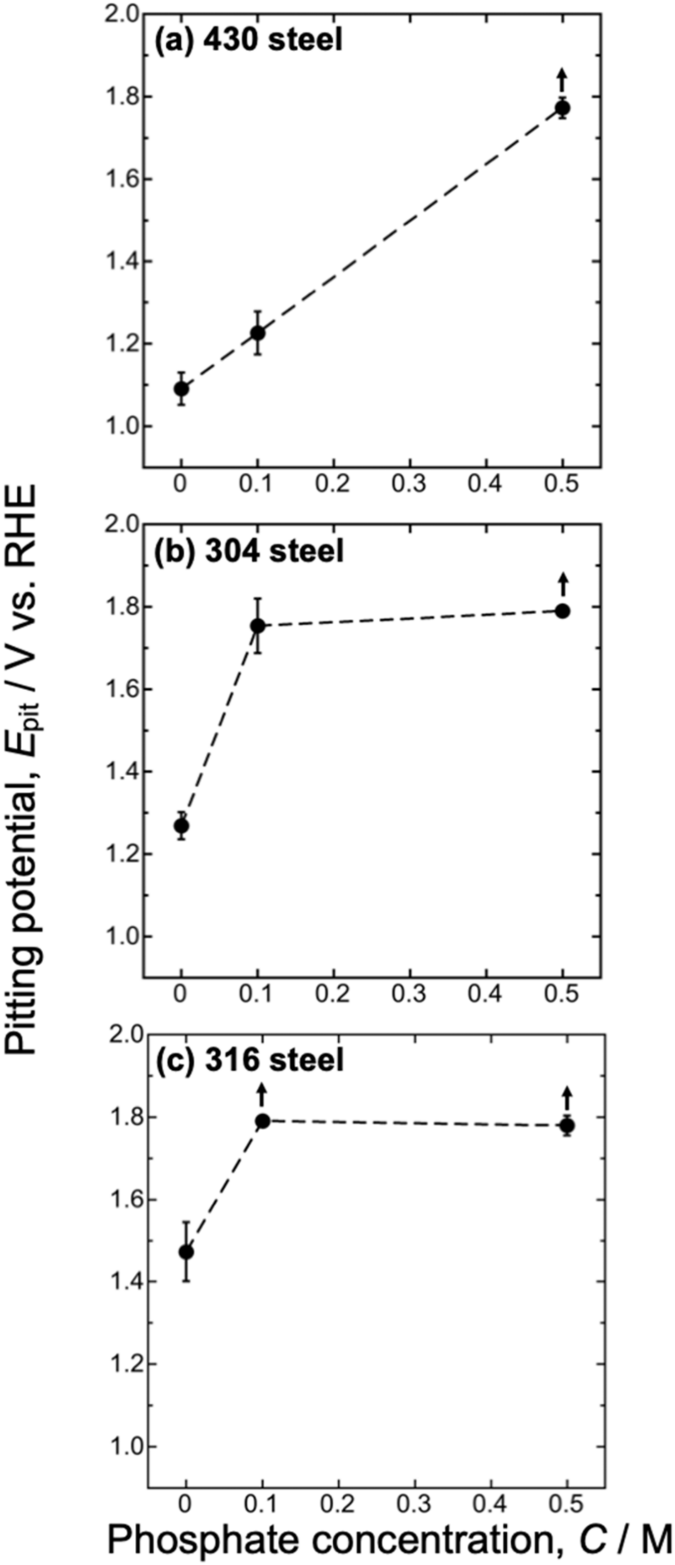
Pitting potentials for (a) 430, (b) 304, and (c) 316 steels as a function of the phosphate concentration in the electrolyte. No pitting corrosion was observed in Figure 2 for the samples marked with arrows.

Overall, these results indicate that adding phosphate to the electrolyte enhances the pitting corrosion resistance of stainless steels. However, when 0.5 M phosphate was added to the electrolyte, a mild increase in the anodic current was observed at higher potentials of approximately 1.2–1.4 V_RHE_. This phenomenon was observed for every steel type and was particularly pronounced in the 430 steel. For the 304 and 316 steels, this peak was absent in the electrolyte without phosphate; however, it became apparent following the addition of 0.5 M phosphate. Therefore, although the addition of phosphate enhanced the pitting corrosion resistance, excessive phosphate appears to promote uniform corrosion at high potentials. The phenomena observed at high potentials are discussed in detail in the following section.

### Phosphate-Induced Changes in the Passive Film Properties: Incorporation of P in the Passive Film

As discussed in the previous section, adding phosphate to the electrolyte enhanced the pitting corrosion resistance of the 430, 304, and 316 steels. Therefore, the subsequent investigation focused on the mechanism underlying this phenomenon. In this study, the mechanisms of enhanced pitting corrosion resistance were considered in terms of changes in the passive film properties, discussed in this subsection, and the pH-buffering effect of phosphate, discussed in the next subsection.

In a previous study, we determined that when phosphate is added to an electrolyte similar to that in this study, P is incorporated into the passive films on Ni, which enhances their protective ability.^19^ Therefore, we predicted that P would also be incorporated into the passive films on stainless steels. XPS was used to verify this prediction. The specimens were held in the electrolytes at either 0.94 or 1.5 V_RHE_ for 1 h. The former potential corresponds to the passive region of the potentiodynamic polarization curves shown in Figure 2a–c, where passive films are expected to form sufficiently on the specimen surfaces. The latter potential corresponds to the region where the peak was observed in the electrolyte with 0.5 M phosphate. After 1 h, the specimens were removed from the electrolyte and analyzed using XPS.

For the as-polished samples, no peaks were observed in the P 2p region. By contrast, after polarization, all the samples showed P 2p peaks. Figure 5 shows the XPS spectra of the P 2p region for the 430, 304, and 316 steels after polarization in the electrolyte containing 0.5 M phosphate at 0.94 V_RHE_. Peaks were observed at 133.2 eV, which were attributed to phosphates.^30,31^ No additional peaks corresponding to other P species were observed. Similar spectra were observed for the 430 steel polarized at 1.5 V_RHE_. These results indicate that P was incorporated into the passive film as phosphate species, which suggests that chromium phosphate, iron phosphate, and their composites had formed.

**Figure 5:**
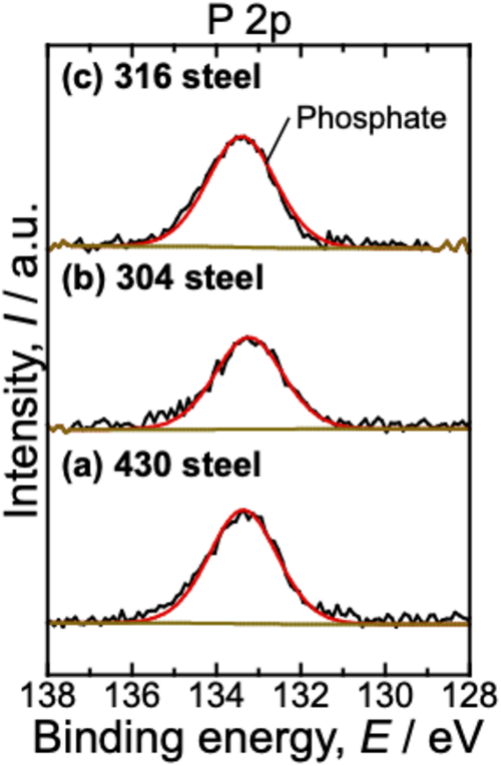
P 2p XPS spectra of (a) 430, (b) 304, and (c) 316 steels after polarization in an electrolyte with 0.5 M phosphate at 0.94 V_RHE_. The peak was attributed to phosphates.^30,31^

Figure 6 shows the concentrations of P in the passive films on the 430, 304, and 316 steels. The results for the 430 steel polarized at 0.94 V_RHE_ (Figure 6a) show that the specimens polarized in the electrolyte containing 0.5 M phosphate exhibited higher P concentrations than those polarized in the electrolyte without phosphate. Furthermore, the specimen polarized at 1.5 V_RHE_ in the electrolyte with 0.5 M phosphate showed a higher P concentration than that polarized at 0.94 V_RHE_. These findings indicate that P was incorporated into the passive film when phosphate was present in the electrolyte. Notably, a trace amount of P was detected in the specimens polarized in the electrolyte without phosphate, which was attributed to phosphate contamination in the electrochemical cell. For the 304 and 316 steels (Figure 6b,c, respectively), the specimen polarized in the electrolyte containing 0.5 M phosphate also exhibited higher P concentrations than those polarized in the electrolyte without phosphate, which confirmed that P was incorporated into the passive film, similar to the case for the 430 steel. These results suggest that the incorporation of P into the passive film and its presence in the passive film as phosphate are independent of whether the steel is ferritic or austenitic.

**Figure 6:**
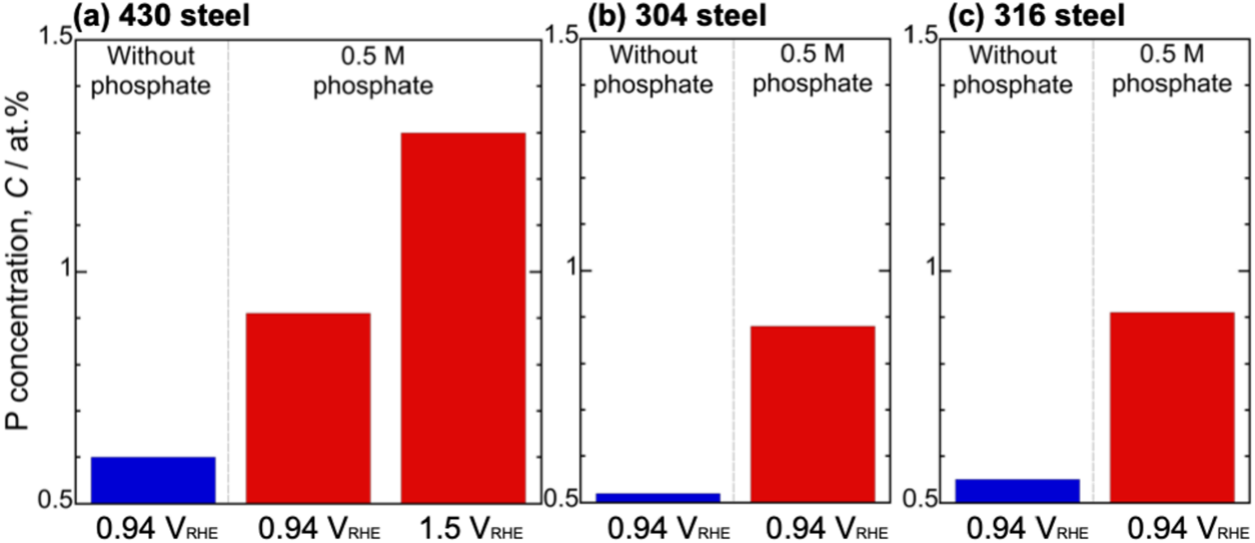
Concentration of P in the passive film on the (a) 430, (b) 304, and (c) 316 steels after potentiostatic anodic polarization in the electrolyte without phosphate and with 0.5 M phosphate at 0.94 or 1.5 V_RHE_ for 1 h.

According to previous studies, the presence of phosphate in the passive films on stainless steel enhances their protective ability. For example, Salah et al. performed impedance measurements on phosphate-containing passive films formed on Sanicro28 stainless steel using industrial phosphoric acid. They reported that the phosphate inhibited the dissolution of the steel surfaces.^20^ It can also be considered that P in passive films may partially dissolve into the electrolyte to form phosphate, which acts as a buffering agent to suppress local pH decrease and could thereby suppress pit propagation. Thus, we can conclude that the phosphate-containing passive films formed on the steels owing to polarization in the electrolyte containing phosphate, and they can be considered to increase the pitting corrosion resistance of 430 steel. However, to fully clarify the mechanism by which P in the passive film enhances pitting corrosion resistance, it is necessary to evaluate the corrosion resistance of passive films formed in a phosphate-containing electrolyte by subsequently exposing them to a phosphate-free electrolyte. These results will be reported in due course.

### Phosphate-Induced Changes in the Passive Film Properties: Decreased Cr/Fe Ratio during Polarization

As described in the previous section, “Effects of Phosphate Addition on Pitting Corrosion Resistance of Stainless Steels,” and shown in Figures 2 and 4, adding phosphate to the electrolyte enhanced the pitting corrosion resistance. However, the results also suggested that polarization at high potentials (approximately 1.2–1.4 V_RHE_) may promote some corrosion, as indicated by the observed current peak, which was most pronounced in 430 steel. This phenomenon was also investigated by analyzing the 430 steel using XPS.

Figure 7 shows the Fe 2p_3/2_, Cr 2p_3/2_, and O 1s XPS spectra for the 430 steel after polishing and polarization at 0.94 and 1.5 V_RHE_ with 0.5 M phosphate. As shown in Figure 7a,b, Fe and Cr metal peaks were observed at 706.8 and 574.1 eV, respectively.^32^ These peaks were observed under all the investigated conditions. Therefore, they were attributed to the bulk of the steel and indicated that the passive film thickness was less than the limit of the XPS detection depth (∼10 nm) under the study conditions. The thicknesses of the passive films are summarized in Table 2, along with the Cr/Fe and O^2–^/OH^–^ ratios in the passive films. The thickness of the passive film increased as the applied potential increased, which suggests that the passive film continued to grow in the high potential range.

**Figure 7:**
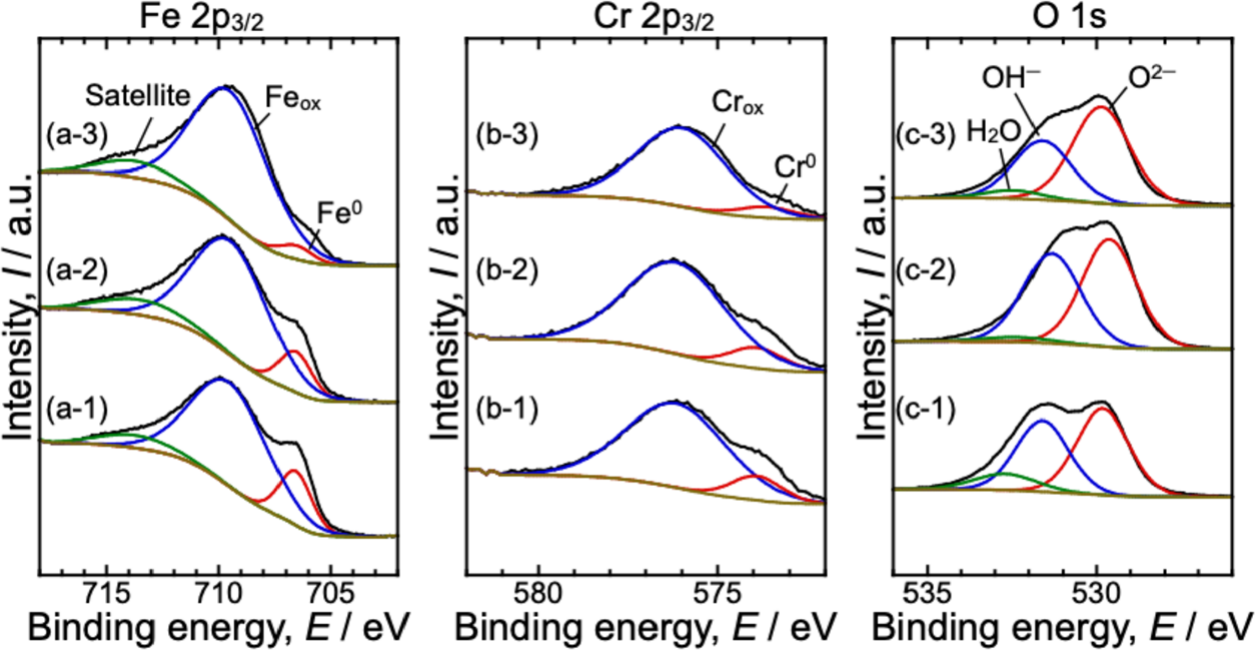
(a) Fe 2p_3/2_, (b) Cr 2p_3/2_, and (c) O 1s XPS spectra for the 430 steel. Spectra were collected in the (1) as-polished state and after polarization in the electrolyte at (2) 0.94 V_RHE_ and (3) 1.5 V_RHE_ with 0.5 M phosphate.

**Table 2:** Thickness, Chromium and Iron Ratios, and Oxide and Hydroxide Ion Ratios in the Passive Films of the 430, 304, and 316 Steels before and after Polarization in Electrolytes without Phosphate and with 0.5 M Phosphate

specimen	electrolyte	condition	thickness [nm]	[Cr]/[Fe]	[O^2–^]/[OH^–^]
430 steel	-	As-polished	5.1	0.19	1.2
	0 M Phosphate	Polarized at 0.94 V_RHE_	6.0	0.16	1.4
	0.5 M Phosphate	Polarized at 0.94 V_RHE_	5.6	0.18	1.6
	0.5 M Phosphate	Polarized at 1.5 V_RHE_	7.4	0.13	1.1
304 steel	-	As-polished	4.8	0.31	1.6
	0 M Phosphate	Polarized at 0.94 V_RHE_	5.2	0.27	2.1
	0.5 M Phosphate	Polarized at 0.94 V_RHE_	4.7	0.43	1.8
316 steel	-	As-polished	4.6	0.23	1.6
	0 M Phosphate	Polarized at 0.94 V_RHE_	5.3	0.29	2.0
	0.5 M Phosphate	Polarized at 0.94 V_RHE_	5.5	0.23	1.9

In general, increasing the polarization potential leads to the enrichment of Cr in the passive film when the potential is below the transpassive dissolution potential for Cr, which results in a higher Cr/Fe ratio. However, for 430 steel, as shown in Table 2, although the Cr/Fe ratio after polarization at 0.94 V_RHE_ in the electrolyte with 0.5 M phosphate was approximately the same as that of the as-polished sample, it decreased at 1.5 V_RHE_. This indicates that the passive film did not simply thicken at higher potentials; rather, Cr was dissolved from the passive film or Fe oxidized preferentially over Cr within this potential range, thereby reducing the Cr concentration in the passive film.

The changes in the O^2–^/OH^–^ ratio further support the theory that the passive film did not simply continue to grow at higher potentials. For stainless steels, anodic oxidation under an applied positive voltage typically results in growth and thickening of the passive film, and metals such as Fe and Cr within the film become more readily oxidized. Consequently, the oxide (O^2–^) concentration in the passive film increases relative to the hydroxide (OH^–^) concentration, resulting in a higher O^2–^/OH^–^ ratio. However, although the O^2–^/OH^–^ ratio increased after polarization at 0.94 V_RHE_ compared with the as-polished specimen owing to metal oxidation, it decreased at 1.5 V_RHE_ and was approximately equivalent to that of the as-polished sample. This decrease was attributed to the dissolution of the components that formed during polarization in the high potential range.

Based on these results, the anodic current peak observed in Figure 2 can be attributed to the partial dissolution of the Cr components from the passive film in the electrolyte with a high phosphate concentration. Previous studies have shown that chromium phosphates, such as CrPO_4_, form in phosphate-containing solutions and may dissolve at higher potentials. Yang et al. reported that phosphate species such as CrPO_4_ could accumulate in the passive film in contaminated phosphoric acid environments; however, these phases are structurally unstable and tend to dissolve as the potential or temperature increases.^30^ Karimi et al. conducted potentiodynamic polarization measurements of a CoCrMo alloy in a phosphate-containing electrolyte (pH 7.4) and reported that phosphate–chromium complex formation and dissolution occurred at potentials of approximately 1.2 V_RHE_.^33^ This is similar to the potential region observed in our study. In this potential region, chromium­(III) can be oxidized to chromium­(IV), which causes the dissolution of the passive film.^34^ Therefore, we conclude that partial dissolution of the Cr components also occurred in the 430 steel. Nevertheless, the amount of Cr dissolved was not quantified directly in this study. Further research is required to fully elucidate the dissolution phenomena under excessive phosphate concentrations for practical applications in seawater electrolysis systems.

### Suppression of Pit Propagation by the pH-Buffering Effect of Phosphate

XPS revealed that P was incorporated into the passivation film, which is considered to be a factor contributing to the enhanced pitting corrosion resistance of stainless steels. However, other factors are also likely to contribute to this improvement. Pitting corrosion consists of two phases: initiation and propagation. Changes in passive films are generally associated with the initiation phase. In addition, the pH-buffering effect of phosphate contributes to the suppression of pit propagation.

The corrosion environment in this study (pH 9.2) is generally considered to be a condition under which stainless steels can stably maintain their passive films. However, when pitting corrosion occurs, the inside of the pit becomes acidified owing to the hydrolysis of metal ions (e.g., Fe^2+^ + 2H_2_O → Fe­(OH)_2_ + 2H^+^), which promotes the passive–active transition. Moreover, acid environments facilitate subsequent active dissolution, leading to pit propagation. Furthermore, phosphate in the electrolyte undergoes equilibrium reactions involving H^+^, and various forms may be present depending on the pH. When the pH is approximately neutral, phosphate ions buffer the solution through equilibrium between H_2_PO_4_^–^ and HPO_4_^2–^, which can release or absorb protons (H_2_PO_4_^–^ ⇌ HPO_4_^2–^ + H^+^). At approximately pH 2, buffering occurs via equilibrium between H_3_PO_4_ and H_2_PO_4_^–^ (H_3_PO_4_ ⇌ H_2_PO_4_^–^ + H^+^). Therefore, phosphate is expected to suppress acidification inside the pits, even after the initiation of pitting corrosion, thereby inhibiting pit propagation. When pitting corrosion occurs, acidification within the pits is suppressed by the pH-buffering effect of the phosphate in the electrolyte. This inhibits pit propagation and facilitates repassivation.

The pit propagation rate after initiation and the repassivation potential were analyzed by conducting cyclic polarization measurements in the electrolyte without phosphate and with 0.1 M phosphate. Figure 8 shows the cyclic polarization curves for the 430 steel measured at room temperature. The anodic sweep results were consistent with those shown in Figure 2a, indicating the occurrence of pitting corrosion. When the current density reached 1 mA cm^–2^, the potential sweep was reversed toward the cathodic direction. At both phosphate concentrations, during the cathodic sweep, the anodic current initially increased, then decreased owing to pit repassivation, and finally, a cathodic current was observed. Once pitting corrosion occurs, its propagation is facilitated by acidification within the pit. Therefore, the increase in the anodic current observed after reversing the potential sweep corresponded to pit propagation, that is, metal dissolution.

**Figure 8:**
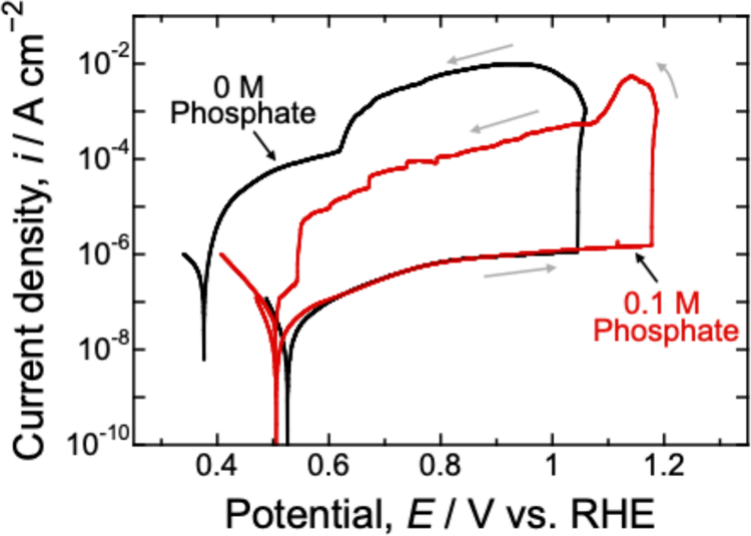
Cyclic polarization curves for the 430 steel in the 0.5 M K-borate–0.5 M KCl electrolyte without phosphate (black line) and with 0.1 M phosphate (red line) at pH 9.2. The measurements were performed at room temperature. The gray arrows in the figure indicate the potential sweep direction.

A comparison of the results revealed that this increase in the anodic current following pit initiation was suppressed in the electrolyte containing 0.1 M phosphate. The total charge during the cathodic sweep, which mainly corresponds to the amount of dissolved metal, was significantly lower in the electrolyte with 0.1 M phosphate than in the electrolyte without phosphate, as shown in Table 3. Furthermore, the repassivation potential at which the current density returned to the cathodic region was higher in the electrolyte with 0.1 M phosphate, which indicated that repassivation of the pits occurred more readily in the electrolyte containing phosphate.

**Table 3:** Total Charge during Cathodic Polarization of 430 Steel Following Pit Growth in Electrolytes without Phosphate and with 0.1 M Phosphate

	phosphate concentration, *C*/*M*	
	0	0.1
total charge, *Q*/*C*	7.25	1.28

Figure 9 shows optical microscopy images of the pits on the 430 steel samples after cyclic polarization and the corresponding depth profiles in electrolytes without phosphate and with 0.1 M phosphate. No significant changes in the size of the pits on the surface were observed; however, the pit depth in the absence of phosphate was approximately 160 μm, which was greater than the pit depth in the electrolyte containing 0.1 M phosphate (approximately 80 μm). The pit depth is strongly affected by the environment inside the pits because a reduction in the pH caused by the hydrolysis of dissolved metal ions promotes pit propagation. Therefore, these results suggest that pit propagation was suppressed by the pH-buffering effect of the phosphate in the electrolyte.

**Figure 9:**
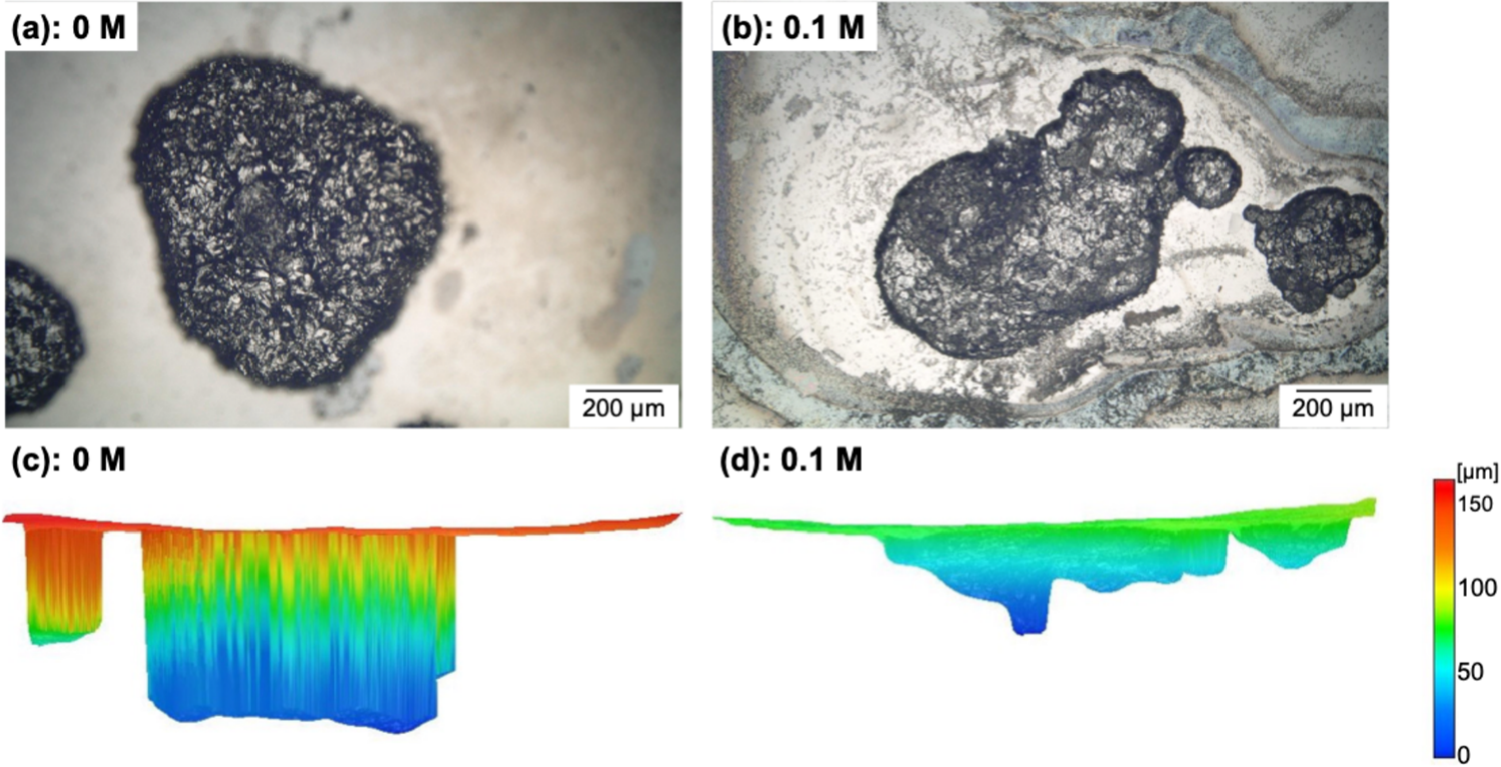
Optical microscopy images of pits on the 430 steel samples after cyclic polarization in the electrolyte (a) without phosphate and (b) with 0.1 M phosphate. (c, d) Pit depth profiles corresponding (a, b), respectively.

These results indicate that, even if pit initiation occurred, pit propagation was suppressed in the electrolytes containing phosphate. As mentioned previously, pit propagation is predominantly attributed to acidification inside the pits. Therefore, the suppression of acidification owing to the pH-buffering effect of phosphate is likely to inhibit pit propagation.

These results demonstrated that phosphate addition enhances the corrosion resistance of stainless steels. In this study, the experiments were conducted at room temperature and with limited ions to investigate the fundamental and intrinsic effect of phosphate on the corrosion behavior of stainless steels. However, in practical seawater electrolysis systems, the operating temperature would be higher, and the electrolytes would contain various other ions. Although it is reasonable to expect that phosphate can impart high corrosion resistance even under such practical conditions, its effectiveness may be more limited. Furthermore, excessive phosphate can induce partial dissolution of Cr components at higher potentials, necessitating consideration of optimal dosage while also accounting for other effects such as energy efficiency including the HER and OER activity. More comprehensive electrochemical analyses, such as long-term wet/dry cyclic tests and electrochemical impedance spectroscopy, is necessary to elucidate the practical long-term stability of stainless steels induced by phosphate addition. These issues will be addressed in detail in future research.

## Conclusions

This study investigated the effects of adding phosphate to an electrolyte containing a large amount of Cl^–^ on the corrosion resistance of stainless steels, particularly ferritic and austenitic steels, for applications in seawater electrolysis systems. The main findings are as follows.

(1) Adding 0.1 M phosphate to the electrolyte enhanced the pitting corrosion resistance of 430, 304, and 316 steels. By contrast, an excessive phosphate (0.5 M) induced the partial dissolution of Cr components at higher potentials, especially for the 430 steel.
(2) XPS analysis showed that P was readily incorporated into the passive films of all the investigated steels (430, 304, and 316) after polarization in the electrolytes containing phosphate, which indicates the addition of phosphate increases the pitting corrosion resistance.
(3) Cyclic polarization measurements demonstrated that the buffering effect of phosphate effectively suppressed pitting propagation in 430 steel.

Therefore, adding phosphate to the electrolyte increases the pitting corrosion resistance of these types of steel, including ferritic steel, which suggests that they may be suitable for applications in the components of seawater electrolysis systems.
